# Itch Beyond the Skin—Mucosal Itch

**DOI:** 10.3389/falgy.2021.700368

**Published:** 2021-10-28

**Authors:** Olivia J. Ly Lesslar, Peter K. Smith

**Affiliations:** ^1^LifeSpan Medicine, Los Angeles, CA, United States; ^2^Cingulum Health, Sydney, NSW, Australia; ^3^Clinical Medicine, Griffith University, Southport, QLD, Australia

**Keywords:** itch, mucosa, neurophysiology, allergy, hypersensitivity, TRP channels, pruritus

## Abstract

Itch is a nociceptive sensation linked with reflexes and cognitive motor actions. We traditionally think of itch as a sensation of the skin related to allergy, an insect sting or interestingly, anxiety and frustration. Less understood and considered are the physiological processes involved in the itching sensation that occurs at mucosal and junctional dermal sites, which is extraordinary as from an evolutionary point of view these sites serve important guardian roles, rich in sensory nerves and inflammatory cells. Despite itch being an ancient reflex and evolutionarily conserved phenomenon, better clinical understanding of the nuances between sites of itch sensation may lead to improved clinical outcomes. This review invites readers to appreciate itch beyond the skin by highlighting several specific itch patterns—nasal, oral, auricular, vulvovaginal, anal, and perineal itch—the pathophysiological mechanisms that underlie them, the clinical patterns these may cause, and some unique treatments.

Most of the literature on the mechanisms and mediators involved in itch is based on our understanding of itch in the skin, also known as pruritoceptive itch, which is generated in the skin by pruritogens either through inflammation *via* various chemical mediators or skin damage (1, 2). Itch is detected by nociceptors and free nerve endings, which in turn excite unmyelinated C-fibers and thinly myelinated Aδ nerve fibers (3). Of the C-fibers there are histamine-dependent and histamine-independent pruritogenic fibers (4). Ongoing research suggests co-signaling of pain and itch sharing the same sensory fibers as well as specific itch-signaling sensory fibers, and it is plausible that both mechanisms exist (1). Neurosensory signals may involve the well-known axon reflex arc, including signaling *via* the dorsal root ganglion, in addition to local activation and local release of neuropeptides, which is known as the antidromic reflex. The site of itch stimulates local neuropeptide release and can activate neurosensory signals that communicate with the somatosensory and motor cortex, leading to itch localization and cognitive awareness of the itch stimuli (1, 3). Referred itch occurs when overlapping sensory distribution of nerves or the pathways of nerve activation is coupled with depolarization of the nerve (5, 6).

The transient receptor potential (TRP) channels comprise 28 members in mammals and 27 in humans that are categorized based on amino acid sequence homology, including TRPA, TRPM, and TRPV (7). Pruritogens either directly or indirectly cause activation of either TRPA1 or TRPV1 ion channels in most cases, or less reported *via* TRPV4, to lower the activation threshold of itch fibers to generate an action potential (8). Many pro-inflammatory compounds from epithelial, neuronal, and inflammatory cells (particularly mast cells, eosinophils, and CD4 lymphocytes) act *via* their respective G-coupled protein receptors, in combination with factors in the local environment, to lower the activation threshold of TRP channels in the genesis of itch (9, 10).

Histamine is an archetypal itch mediator, activated *via* a G-coupled protein to reduce the activation threshold of an ion channel: TRPV1, which in turn results in calcium influx and release of neuropeptides from sensory nerve fibers (11). Itch is clearly more complex than histamine alone. Histaminergic itch alone is also complex, with H1 and H4 antagonists reducing some components of itch, whereas H3 receptor antagonists provoke itch (12, 13).

“Priming” is a phenomenon whereby repeated exposure to an allergen leads to a response being elicited by less of that allergen, other allergens, or even non-allergic stimuli (irritants). Sensory nerves release neuropeptides that induce chemotaxis, activation, proliferation, and survival factors for leukocytes, particularly eosinophils (9). Eosinophils are the main source of nerve growth factor in the body and may have a trophic effect on increasing neuroreceptors, growth of sensory nerves and neurites (14). Locally released nerve factors, such as calcitonin gene-related peptide and substance-P, promote eosinophil migration, activation, and survival, creating a potential amplification loop for itch (15).

Mucosal junctional sites to skin such as the eyes, nose, mouth, anus, and vagina are highly innervated, owing to these areas being guardian sites where the tight junctions regulate the passage of ions and macromolecules through the paracellular pathway (16). Unchecked disease processes at these sites may easily result in an amplification loop and increased nerve density, driven by itch and release of inflammatory mediators (including histamine, neuropeptides, and eosinophils).

## Nasal, Ocular and Auricular Itch

Itch is a hallmark of many forms of rhinitis, and increased expression of trigeminal sensory nerves has been noted in the nasal epithelium of patients with chronic allergic rhinitis (17–19). There is also an increased expression of TRPV1 ion channels in non-allergic rhinitis (20). The ophthalmic nerve and maxillary nerve are both terminal branches of the trigeminal nerve, which innervate the oral cavity, the lumen of the upper airways, and the conjunctiva of the eyes, each being limited by epithelial tight junctions (21). They respond to a wide range of chemicals, usually at concentrations about three orders of magnitude higher than many olfactory responses (22). In the presence of inflammation, allergen source-derived proteases destroy tight junctions in the airways, exposing sensory nerve fibers to irritants, creating an environment for a lowered activation threshold of sensory nerves fibers (16).

Studies have identified patients with allergic rhinoconjunctivitis as having itch of the nose and eyes as major symptoms of this disease, however itching of the throat, palate, and ear canal are also common symptoms contributing to the disease burden (23–25). Itch of the nose and eyes may appear to be a simple response to allergen exposure at these sites, however there are also complex naso-ocular reflexes that occur, where ocular itch can be elicited by nasal provocation and improved by treatments delivered into the nose (26).

**Figure 1:**
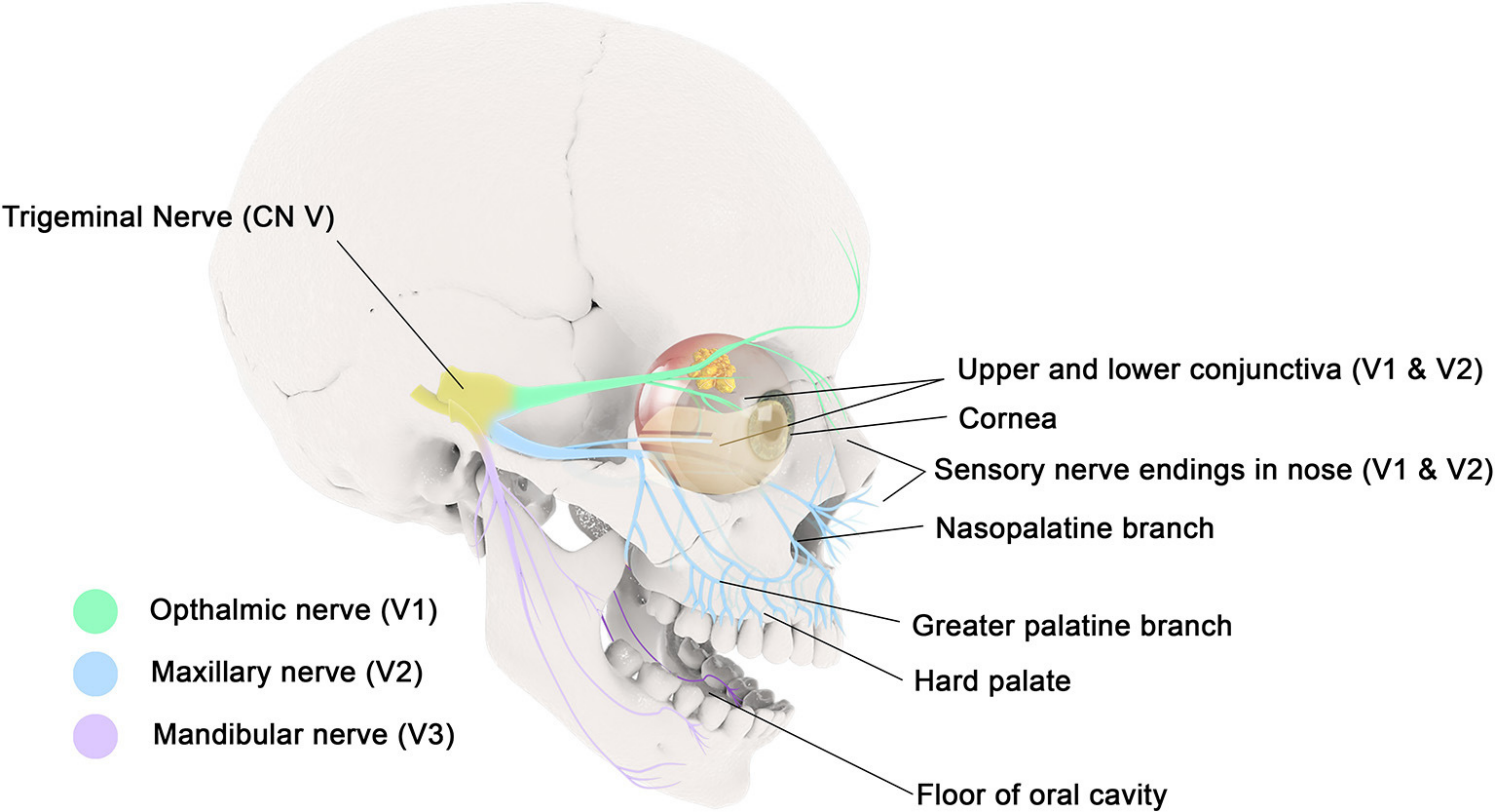
The ophthalmic nerve (V1) innervates the mucous membranes of the frontal and ethmoidal sinus, upper eyelid and its conjunctiva, and cornea. The maxillary nerve (V2) innervates the mucous membranes of the lower eyelid and its conjunctiva, maxillary sinus, nasal cavity, upper lip, upper gingiva, and hard palate. The mandibular nerve (V3) innervates the mucous membranes of the floor of the oral cavity, the lower lip, anterior two-thirds of the tongue, and lower gingiva.

The spinal trigeminal nucleus incorporates sensory information from different cranial nerves, including the trigeminal nerve, facial nerve, glossopharyngeal nerve, and vagus nerve (27); thus there may be some loss of fidelity as to the source of irritation which may contribute to referred itch. The ophthalmic nerve (V1) innervates the skin and mucous membranes of the forehead and scalp, frontal and ethmoidal sinus, upper eyelid and its conjunctiva, cornea, and dorsum of the nose. The maxillary nerve (V2) innervates the skin and mucous membranes of the lower eyelid and its conjunctiva, cheeks and maxillary sinus, nasal cavity and lateral nose, upper lip, upper teeth and gingiva, and superior palate. The mandibular nerve (V3) innervates the skin and mucous membranes of the floor of the oral cavity, the lower lip, anterior two-thirds of the tongue, lower teeth and gingiva (Figure 1). The vagus nerve (X) innervates the external canal of the ear and the internal surfaces of the laryngopharynx and larynx.

Peripheral neuro-inflammation can be provoked by sensory nerve activation, however it may be possible for reflex arcs or central perception of itch to be also involved (28). Itch of the ear is a bothersome symptom in patients with rhinitis. While the vagus nerve has a small cutaneous distribution that innervates the external ear canal, referred itch from other cranial nerves innervating the mouth or nasopharynx may be causal. In a similar manner, itch of the hard palate is perceived by the greater palatine and nasopalatine nerves, both of which are branches of the maxillary division of the trigeminal nerve (Figure 1).

In a double-blind, randomized-controlled trial with a series of patients diagnosed with non-allergic rhinitis challenged with hyper-osmolar mannitol nasal spray, many patients experienced itch in extra-nasal sites: 45% in the throat, 22% in the palate, 27% in the auricular canal, and 20% in the eyes. What was fascinating clinically was the almost instant provocation of referred itch, and their rapid (within 5 min) relief with use of 0.6% olopatadine nasal spray which suggests sensory mechanisms at play (29).

## Oral Itch

Oral itch of the hard and soft palate, as outlined above, can occur with rhinitis. In the context of allergy, one of the most common forms of oral itch is oral allergy syndrome (OAS) which is also known as the “pollen-food allergy syndrome” occurring in up to 8% of patients with inhalant pollen allergy (30). When a patient is sensitized to inhalant pollen allergens, ingestion of fruit, vegetables or nuts that contain similarly structured proteins to the pollens that induce an immunoglobulin E (IgE) reaction involving mast cells in the oropharynx to provoke intense itch in the mouth. Many of these plant allergens belong to the profilin family and are rapidly degraded by digestive processes or cooking and can be controlled with antihistamines, however, it is also possible for anaphylaxis to occur in patients with OAS (31). It is also worth noting the OAS can occur with dust mite allergy when ingesting crustacea due to cross reacting arthropod allergens (32).

Related to OAS is the latex-food syndrome or latex-fruit allergy where, for example, a banana or kiwi allergy may be connected to a latex allergy because these have structurally similar epitopes recognized by IgE. This cross-reactivity may mean that patients who develop a latex allergy, may develop itch in the mouth when exposed to an increasing number of plant sources, such as avocado, banana, chestnut, kiwi, peach, tomato, potato, and bell pepper. Clinicians need to be aware that the two may be related and, it is not always clear whether latex sensitization precedes or follows the onset of food allergy (33).

## Vulvovaginal Itch

The role of sensation in the vulvovaginal area has protective and sexual functions. Certain physiologies (e.g., acidosis, an elevation in core body temperature), irritation or disease states can provoke intense itch of these highly sensory-innervated tissues (34, 35). Tissue damage through excoriation or inflammation can evoke further downstream pro-inflammatory effects *via* the purinergic signaling cascade which involves extracellular breakdown products of adenosine triphosphate (ATP) activating purinergic receptors (36). The primary nerves involved in somatosensory responses of the external vulva are the perineal nerve and the dorsal nerve of the clitoris, whereas the internal vulva and cervix have sensory fibers that coalesce in the paracervical-uterovaginal plexus. Interestingly, it was only relatively recent that active exploration of the distal course of the dorsal nerves of the clitoris and associated structures was undertaken. A 2020 anatomical dissection study stated that the clitoral body is substantial in length (mean = 37.0 mm), mostly lying superficially under the clitoral hood and mons pubis (in nine of the 10 cadavers, the dorsal nerves could be traced to within 6.0 mm of the glans). The dorsal nerves of the clitoris were also noted to be larger than previously recognized; ranging from 2.0 to 3.2 mm in diameter on average, along their course in the clitoral body, terminating at or near the base of the clitoral glans (37). Animal studies suggest an increase in TRPA1 sensory receptors following vulvovaginal inflammation in early life, indicating neural and hyperalgesic plasticity. This highlights the need for clinical awareness of new insights into clitoral anatomy, and providing swift and appropriate treatment, especially in the pediatric population (38).

Mucosal hyperinnervation with nociceptors has been demonstrated by repeated vaginal candida albicans infections (39). Candida yeast infection affects at least 75% of women at least once in their lifetime. Candidiasis is the most common cause of lower genital tract pruritus and discomfort, involving pain, burning and itch (40). The cell wall of candida albicans is composed of β-glucan which can activate both TRPA1 and V1 pathways as well as *via* purinergic pathways (41).

The TRPA1, TRPV1 and purinergic sensory receptors are hormonally responsive (42–44) which may account for the periodic nature of cyclic vulvovaginitis. These may refer to a recurrent flare of candidiasis or cytolytic vaginosis which, respectively, occur at specific stages of the menstrual cycle (45). Lack of estrogen, especially in menopause leading to atrophic vaginitis, can also result in itch due to the deterioration in the epithelial barrier (and epithelial and glandular products) that protect sensory nerves, reducing their activation threshold. Not all vulvovaginal itch in the post-menopausal female is due to atrophy though. Clinicians should note that most women with vulvar carcinoma are older than 70 years (46) and a systematic review of 3,322 patients with vulvar intraepithelial neoplasia reported that 64% of these women presented with pruritus or pain (47).

## Anal and Perineal Itch

The anus and perineal tissue have sensory and motor innervation from the pudendal nerve. Approximately 60% of the dorsal root ganglion that receives sensory information from the perineum expresses TRPV1 and ~75% of perineal C-fibers expressing TRPV1 are thought to have some hormonal regulation due to having estrogen receptors (48, 49). The pudendal nerve is a major somatic nerve of the sacral plexus, and innervates the external genitalia of both sexes, the perineum and the skin around the anus so referred itch is common (50).

Pruritis ani is a mucosal itch syndrome occurs in up to 5% of the population and is four times more common in men. Pruritis ani has many triggers including infections (bacteria, fungal, viral, helminths), hemorrhoids and diet (51). A prospective, randomized, placebo-controlled, double blind, crossover trial found that consumption of red-hot chili pepper increased symptoms of pain and anal itch (52). In fact, capsaicin has been used clinically to treat this condition by desensitizing TRPV1 function in the anus (53). Fungal and viral-mediated itch involve not only TRPV1 but also TRPA1 and purinergic pathways, thus capsaicin may fail to reduce itch arising from these etiologies. Should frontline treatments not provide symptomatic relief, destruction of sensory nerves by way of “anal tattooing” using a mixture of methylene blue dye, local anesthetics, and hydrocortisone has reported efficacy in refractory idiopathic pruritus ani (51, 54).

## Conclusion

Itch is a complex nociceptive response that is present at all mucosal “guardian” sites—regions where the mucosal surfaces meet the external epithelium. These serve an evolutionary benefit to having highly developed sensory mechanisms in these areas, with an increased capacity for inflammation and itch responses to possible threats. However, due to the complexity of the evolved neural networks, an awareness of referred itch patterns is important clinically. Under or poorly treated itch may result in amplification loops and increased symptom burden, emphasizing the need for clinicians to identify the source (and originating site) of pathology early and initiate appropriate treatment.

## Author Contributions

Both authors contributed to the concept, the paper framework, and writing of the paper.

## Conflict of Interest

The authors declare that the research was conducted in the absence of any commercial or financial relationships that could be construed as a potential conflict of interest.

## Publisher's Note

All claims expressed in this article are solely those of the authors and do not necessarily represent those of their affiliated organizations, or those of the publisher, the editors and the reviewers. Any product that may be evaluated in this article, or claim that may be made by its manufacturer, is not guaranteed or endorsed by the publisher.
